# Role of Ectomycorrhizal Symbiosis Behind the Host Plants Ameliorated Tolerance Against Heavy Metal Stress

**DOI:** 10.3389/fmicb.2022.855473

**Published:** 2022-03-28

**Authors:** Eetika Chot, Mondem Sudhakara Reddy

**Affiliations:** Department of Biotechnology, Thapar Institute of Engineering and Technology, Patiala, India

**Keywords:** ectomycorrhizal fungi, heavy metal stress, host plants, metal tolerance, symbiosis, metal defense mechanisms

## Abstract

Soil heavy metal (HM) pollution, which arises from natural and anthropogenic sources, is a prime threat to the environment due to its accumulative property and non-biodegradability. Ectomycorrhizal (ECM) symbiosis is highly efficient in conferring enhanced metal tolerance to their host plants, enabling their regeneration on metal-contaminated lands for bioremediation programs. Numerous reports are available regarding ECM fungal potential to colonize metal-contaminated lands and various defense mechanisms of ECM fungi and plants against HM stress separately. To utilize ECM–plant symbiosis successfully for bioremediation of metal-contaminated lands, understanding the fundamental regulatory mechanisms through which ECM symbiosis develops an enhanced metal tolerance in their host plants has prime importance. As this field is highly understudied, the present review emphasizes how plant’s various defense systems and their nutrient dynamics with soil are affected by ECM fungal symbiosis under metal stress, ultimately leading to their host plants ameliorated tolerance and growth. Overall, we conclude that ECM symbiosis improves the plant growth and tolerance against metal stress by (i) preventing their roots direct exposure to toxic soil HMs, (ii) improving plant antioxidant activity and intracellular metal sequestration potential, and (iii) altering plant nutrient uptake from the soil in such a way to enhance their tolerance against metal stress. In some cases, ECM symbiosis promotes HM accumulation in metal stressed plants simultaneous to improved growth under the HM dilution effect.

## Introduction

The advancements in human technologies such as industrialization and urbanization increase the soil heavy metal (HM) pollution. HMs can also leach down into groundwater or can be transferred to the successive levels of the food chain (Khosla and Reddy, 2014), causing a significant threat to living organisms and the environment (Vaclavikova et al., 2008; Nagajyoti et al., 2010). Both anthropogenic sources (such as industrialization, agriculture, sewage sludge, traffic emissions, and untreated wastewater) and natural sources (such as volcanic eruptions, rock weathering, and windblown dust) can contribute to the soil HM pollution (Srivastava et al., 2017). HMs are mainly categorized as essential or non-essential based on their role in various biological functions such as cell structure stabilization and enzyme catalysis (Bruins et al., 2000). The non-essential HMs are not required for cell metabolisms and are highly toxic for cells even in trace amounts (Haferburg and Kothe, 2007). HM toxicity in plants can reduce plant biomass, seed germination, fruit yield, nutrition content, and root and shoot length and induce chlorosis and mortality (Rai et al., 2021). The plant immune system, production of photosynthetic pigments such as carotenoids, chlorophylls, and reactive oxygen species (ROS) scavenging systems is also predominantly affected in the plants subjected to the high concentrations of HMs (Rastgoo and Alemzadeh, 2011; Ghnaya et al., 2013). HM toxicity can induce oxidative stress in plants (Khator and Shekhawat, 2020), further damaging cellular biomolecules such as protein and nucleic acids (Romero-Puertas et al., 2019). HM stress in plants leads to the impaired growth of primary root and root hairs (Broadley et al., 2007; Hayat et al., 2012; Feleafel and Mirdad, 2013), thus resulting in reduced water uptake efficiency of host plants (Rucińska-Sobkowiak, 2016). The content of HMs uptake varies with plant species and depends on environmental factors such as temperature, pH, nutrients, and moisture. For example, the accumulation of metal ions in *Beta vulgaris* is higher in summers than in winter due to the relatively high transpiration rates (Sharma et al., 2007). From plants, the metal can enter into higher trophic levels of food chains such as insects, herbivores, and humans, resulting in the ecosystem and food chains imbalance (Zhang et al., 2017). HM toxicity reduces the growth of plants in terms of dry weight and height, which can be improved by plant symbiotic association with ectomycorrhizal (ECM) fungi. The transmission of HMs from soil to plants is highly influenced by the presence of ECM fungal partners in the symbiosis with plant roots (Tang et al., 2019; Sun et al., 2021).

Ectomycorrhizal fungi are ubiquitous symbionts of plants, predominately found in Boreal and Temperate biomes. They colonize the roots of a wide range of woody plants such as *Eucalyptus*, *Pinus*, *Acacia*, and *Picea* (Smith and Read, 2010). ECM fungi play a critical role in nutrient dynamics of the terrestrial ecosystem by facilitating the mobilization of soil unavailable nutrients and water to host plants in return to their photosynthesis driven carbon (Smith and Read, 2010; Van der Heijden et al., 2015; Hodge, 2017). ECM fungi possess highly efficient and diverse defense mechanisms against HM stress, allowing them to thrive on metal-polluted lands (Khullar and Reddy, 2018). They enhance the tolerance of host plants against metal stress by various mechanisms and play a critical role in the bioremediation of metal-contaminated lands (Jourand et al., 2014; Reddy et al., 2016; Liu B. et al., 2020).

**FIGURE 1:**
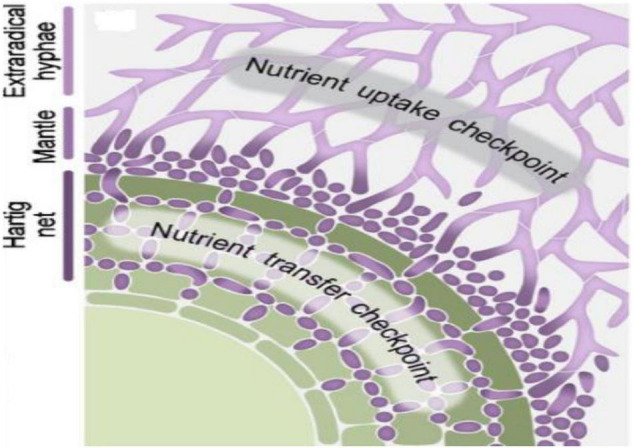
Schematic view depicting various parts (Hartig net, mantle, and extraradical hyphae) of established Ectomycorrhizal (ECM) symbiosis with plant root cells and regulatory checkpoints of nutrient transfer that occurs between ECM fungi and plant in established symbiosis. Reproduced from the work of Garcia et al. (2015) with permission of John Wiley and Sons.

Ectomycorrhizal fungi in symbiosis develop into extramatrical mycelia growing in the soil surrounding the rhizosphere, aggregated fungal hyphae to ensheath lateral roots called as a mantle, and hyphae penetrating the apoplastic zones of cortical and epidermal cells of the host roots named as Hartig net (Figure 1; Martin et al., 2016). The extramatrical hyphae, the potential sinks for host plant-derived carbon, act as an essential candidate for delivering the carbon to the soil. They also play a significant function in N dynamics (Wu, 2011), P uptake (Cairney, 2011), and mineral weathering (Landeweert et al., 2001; Rosling, 2009). The immense networks of ECM fungal mycelia in the soil can also link the root tips of different tree species. The labeled carbon (^13^C) derived from tree *Picea* can transfer to the surrounding trees through ECM mycelia networks, where the exchange is found to be higher with phylogenetically related trees. The ECM communities among the phylogenetically related trees are very similar in composition (Rog et al., 2020). The diversity of ECM fungi is significantly determined by edaphic factors such as (i) soil moisture (Jarvis et al., 2015), pH, carbon, and N and P content (Veach et al., 2018); (ii) type of host (Tedersoo et al., 2014; Saitta et al., 2018), host age (Zhang et al., 2014), and host genotype (Wang et al., 2018); and (iii) environmental factors such as climatic gradients (Steidinger et al., 2020), location coordinates on mountain slopes (Wei et al., 2020), light availability (Kummel and Lostroh, 2011), and canopy and terrestrial soils (Nilsen et al., 2020).

It is well-known fact that ECM fungal symbiosis improves their host plants tolerance against metal stress (Jourand et al., 2010, 2014; Sun et al., 2021) and that ECM symbiosis application in plant regeneration on metal contaminated land has received considerable focus in global research (Wen et al., 2017; Shi et al., 2019; Dagher et al., 2020). Various reports concerning HM defense mechanisms in ECM fungi (Kalsotra et al., 2018; Khullar and Reddy, 2018; Shi et al., 2018, 2020) or plants (Hasan et al., 2017; Li et al., 2019; Schat et al., 2020) are available separately. Thus, understanding how ECM symbiosis affects and regulates the different plant defense mechanisms against metal stress has prime importance. This field is not extensively studied and reviewed but requires more focus to reinforce ECM symbiosis as a bioremediation tool for rehabilitating metal contaminated lands with plants. The present review mainly focused on understanding how ECM symbiosis affects the various host plant defense systems against metal stress, which thus results in their enhanced metal tolerance.

## Ectomycorrhizal Symbiosis–Driven Mechanisms Behind Enhanced Metal Tolerance of Their Host Plants

**FIGURE 2:**
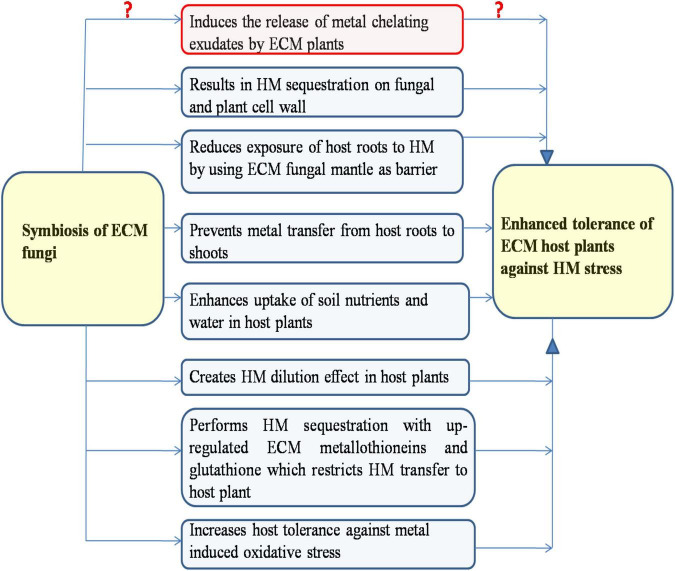
Ectomycorrhizal (ECM) symbiosis mediated different mechanisms conferring their host plants an increased tolerance against heavy metal stress (ECM symbiosis induces or represses the release of organic acids from ECM plants remain unclear).

Plants have diverse molecular and physiological mechanisms to counteract the HM stress, which broadly includes HM exclusion, compartmentalization, chelation, sequestration, and mitigation of HM-induced oxidative stress (Joshi et al., 2019; Zhu et al., 2021). Various plant defense mechanisms against HM stress have been reported to be positively affected by ECM symbiosis for their improved tolerance against HM stress, which are discussed in the following (Figure 2).

### Extracellular Secretion of Organic Acids by Ectomycorrhizal Plants Under Heavy Metal Stress

The assessment of bioavailable content forms a solid basis for predicting soil pollution and risk (Gonzalez-Chavez et al., 2004). Several environmental factors such as soil pH strongly affect the HM availability (Fässler et al., 2010; Bolan et al., 2014; Liu B. et al., 2020), with alkaline soil pH favoring metal unavailability (Fernández-Fuego et al., 2017). ECM-mediated reduction in bio-available or exchangeable soil HM content decreases metal toxicity on their host plants (Shi et al., 2019). The exudates of mycorrhizal fungi contain organic acids such as oxalic acid, succinic acid, malic acid, and formic acid, which chelate the metals and play an important role in metal detoxification (Meharg, 2003; Ray and Adholeya, 2009; Colpaert et al., 2011). The trivalent ions such as Fe^3+^ and Al^3+^ can form strong complexes with chelating agents like oxalate, citrate, and malate (Jones, 1998). The ECM fungal symbiosis induces the secretion of root exudates by host plants, which, in soil, chelates metal ions, thereby reducing their toxicity on plants (Gonzalez-Guerrero et al., 2008; Acosta et al., 2014). Many fungal species generally produce oxalic acid in large amounts (Dutton and Evans, 1996). Higher levels of oxalic acid production were reported in mycorrhizal plants of *Pinus sylvestris* in the presence of Al (Ahonen-Jonnarth et al., 2000). Contrary to this, a lower amount of oxalic acid production was recorded in mycorrhizal plants under high Ni stress, thus suggesting fungal sheath as a barrier restricting HM uptake in ECM roots, leading to reduced HM accumulation (Jourand et al., 2010). Although reduced organic acid production in metal stressed ECM plants relative to non-mycorrhizal plants has been reported, the ECM symbiosis enhances their host plant growth under metal stress (Fernández-Fuego et al., 2017). Plants have to pay high metabolic costs to survive the HM-induced oxidative stress, which could cause the reduced production of organic acids in plants under high HM stress (Iori et al., 2012). In support of this, Fernández-Fuego et al. (2017) showed that ECM-mediated enhanced HM accumulation in their metal stressed host plants reduces organic acid production compared to non-mycorrhizal control and *vice versa*. The utilization of plant high energy in expressing metallothioneins (MTs) (see section “Intracellular Heavy Metal Sequestration With Metal-Chelating Compounds”) reduces the growth of Willow plants upon ECM inoculation in *Betulapubescens* (Lanfranco, 2007). Besides the variations in the conclusion of the different studies described above, Ahonen-Jonnarth et al. (2000) also reported the varying potential of ECM symbiotic *Pinus sylvestris* to produce organic acids under metal stress depending upon the different ECM fungal species, HM type, and its concentration. Other ECM fungal species–based variations suggest their different metal tolerance strategies (Ahonen-Jonnarth et al., 2000; Courbot et al., 2004), which could also be possible due to varying fungal cell wall efficiencies to bind with HMs (Johansson et al., 2008). Hence, on the basis of these contradictory results, the impact of ECM symbiosis on the secretion of plant root exudates under metal stress cannot be precisely commented on and requires more studies.

### Ectomycorrhizal-Associated Plant Roots Against Heavy Metal Toxicity

The ECM fungus, *Paxillus involutus*, inoculated to *Pinus sylvestris* showed the localization of Pb aggregates in epidermis and cortex of roots but not in stem or roots endodermis. These results suggest that roots are significant plant defense operators against metal stress (Bizo et al., 2017). The number of electronegative sites present on the cell wall of fungal mycelium for HM binding and, further, the fungal potential of intracellular HM binding forms the firm basis of varying efficiency of different ECM fungi in providing HM tolerance to their host plants (Shi et al., 2019). HMs binding to the sulfhydryl and phosphate compounds intracellularly or by binding with electronegative sites on fungal cell wall confers that ECM fungi provide high tolerance toward HM stress (Bizo et al., 2017). Several past studies focused on determining the impact of ECM inoculation on HM transfer from soil to host plants had generated diverse and contradictory results from each other. (i) Some studies reported ECM fungi as a physical barrier between soil HM content and plants, thereby reducing metal accumulation in plants (Colpaert and van Assche, 1993; Baum et al., 2006; Reddy et al., 2016). Under increasing Pb stress, ECM plants accumulate comparatively fewer Pb than non-mycorrhizal plants due to the fungal mantle-mediated reduction in roots exposure with Pb, eliminating the requirement of high energy-consuming plant defense metabolism against HM in ECM plants (Szuba et al., 2020). (ii) In contrast, other studies showed an increase of HM absorption and accumulation in plants upon ECM fungal inoculation (Fernández et al., 2008; Wen et al., 2017; Tang et al., 2019). Furthermore, ECM fungi can enhance the HM accumulation in plant roots but alleviate its transport into shoots (Kozdrój et al., 2007; Mrnka et al., 2012; Yu et al., 2020). Zong et al. (2015) reported that the extent of HM uptake by ECM roots and further transfer to shoots varies according to different ECM species inoculated with the same host separately and with other host plants. The soil HMs taken up by mycorrhizal fungi are loaded from their Hartig net to host roots. Still, the restricted apoplastic pathway of root endoderm due to Casparian strips causes the higher HM accumulation in roots than plant other tissues (Luo et al., 2014). (iii) A few studies have reported that ECM fungi could both increase and inhibit HM transport to the host plants (Marschner et al., 1996; Zimmer et al., 2009). In the case of plant nutrient metals such as Zn, the ECM fungi can perform the dual function of promoting and inhibiting HM accumulation in host plants depending upon its low and high toxic concentration present in an external environment, respectively (Zhang et al., 2021). The enhanced uptake of essential and non-essential HM by ECM roots was reported under low HM concentration, whereas the metal uptake got restricted under high HM concentrations, keeping plants healthy in both cases (Bojarczuk and Kieliszewska-Rokicka, 2010; Fernández-Fuego et al., 2017). The variability, in conclusions, derived from various studies could be due to several factors such as differences in culture medium used, period of HM treatment, and mycorrhization rates (Tang et al., 2019). While increasing the HM content of their host plants under HM stress, ECM fungi also improve plant growth, which the HM dilution effect can describe. The inoculation of ECM fungi promotes the nutrients, water, and HM uptake in host plants, which results in improved plant growth and dilution of HM content in metal stressed plants, thus reducing the HM-mediated toxicity in plants (Shi et al., 2019; Tang et al., 2019). Under Cd stress, the ECM plants can couple the net Cd influx with net H^+^ efflux through H^+^-ATPases, causing a higher Cd influx than non-ECM plant roots. Although Cd accumulates higher in ECM plant roots and leaves than non-ECM plants, the improved C assimilation, growth, and nutrition status of ECM plants provide them an enhanced growth compared to non-ECM plants under Cd stress (Table 1; Ma et al., 2014). The ECM inoculation increases the host plant biomass on metal-contaminated land. It improves the phytoextraction of HM in the host plant, resulting in reduced soil content compared to non-mycorrhizal plants (Dagher et al., 2020). To enhance the phytoremediation of HM pollution, the increase in HM mobilization can also lead to HM leaching to groundwater, which thus requires careful monitoring (Bolan et al., 2014).

**TABLE 1:** Ectomycorrhizal (ECM) symbiosis–based altered parameters of metal stressed host plants, which confers them a better HM tolerance than non-ECM plants [upward (↑) and downward (↓) arrows show activity increase and decrease, respectively].

ECM fungi	Host plant	Metal stress	Metal exposure period	Parameters of plants	Activity level in ECM plants relative to non-ECM plants	Conclusions	References
*Pisolithus albus*	*Eucalyptus globulus*	60 mg Ni kg^–^^1^ of substrate	12 weeks	∙ Root Ni content ∙ Shoot Ni content	↓	ECM fungal as barrier in plant HM uptake from soil	Jourand et al., 2010
*Paxillus* *involutus*	*Populus × canescens*	0.75 mM Pb(NO_3_)_2_	6 weeks	∙ Pb root content ∙ ROS burst in roots H^+^ ATPases ∙ Pb sequestration ∙ Cytoskeleton modifications	↓ ↓ ↓ ↓	Pb biofiltering effect by ECM fungi	Szuba et al., 2020
*Paxillus* *Involutus*	*Populus × canescens*	50 μM CdSO_4_	40 days	∙ Cd influx in plant ∙ Plant growth and nutritional status	↑ ↑	Improved host growth under HM dilution effect	Ma et al., 2014
*Pisolithus albus*	*Eucalyptus globulus* and *Acacia spirorbis*	Ultramafic substrate rich in heavy metals	Pre- contaminated site	∙ Uptake of soil deficient essential nutrients ∙ Barrier to toxic metals	↑ ↑	Increases growth by enhanced nutrient uptake and metal avoidance	Jourand et al., 2014
*Pisolithus* sp.	*Pinus thunbergii*	100–800 mg Cr kg^–^^1^ of soil	5 months	∙ Percentage content of bioavailable or exchangeable Cr in soil	↑	Relieving HM toxicity on host plants	Shi et al., 2019
*Pisolithus albus*	*Eucalyptus tereticornis*	150 μM Cu 40 μM Cd	4 weeks	∙ Metal uptake in host plant	↓	Metal immobilization in fungal extraradical mycelium by upregulated fungal metallothionein	Reddy et al., 2016
*Suillus luteus*	*Quercus acutissima*	0.1 mg/kg and 5 mg/kg Cd	14 days	∙ Catalase ∙ MDA content ∙ Glutathione	↑ ↓ ↑	Mitigation of metal-induced oxidative stress by ECM symbiosis	Sun et al., 2021
*Paxillus ammoniavirescens*	*Betulapubescens*	Multi metal stress	Pre-contaminated soil samples	∙ Production of antioxidants ∙ Host plant biomass ∙ Metal accumulation in host	↑ ↑ ↓	Enhanced tolerance to oxidative stress and barrier to HM uptake	Fernández-Fuego et al., 2017
*Pisolithus tinctorius*	*Eucalyptus*	1,000 μM Mn	90 days	∙ Mg content	↑	Metal dilution effect	Canton et al., 2016
*Suillus luteus*	*Pinus massoniana*	0.4 mmol L^–1^ Al	59 days	∙ Malondialdehyde content (indicator of oxidative stress)	↓	Reduction in HM induced oxidative stress	Liu H. et al., 2020
*Sphaerosporella brunnea*	*Salix miyabeana*	Decommissioned industrial land (Multimetal stress)	Pre-contaminated industrial plots	∙ Host plant biomass ∙ Soil Cu, Pb, and Sn content after ∼4 years of inoculation	↑ ↓	Enhanced plant growth and phytoextraction under metal stress	Dagher et al., 2020

### Intracellular Heavy Metal Sequestration With Metal-Chelating Compounds

Metallothioneins are cysteine-rich low–molecular weight proteins that strongly bind to metal ions through their thiol groups of cysteine residues, thus playing an essential role in metal sequestration and detoxification (Cobbett and Goldsbrough, 2002; Zhu et al., 2009). Besides the metal chelation, plant MTs function in scavenging accumulated ROS under oxidative stress (Xue et al., 2009: Hu et al., 2011; Ansarypour and Shahpiri, 2017; Malekzadeh and Shahpiri, 2017). The plant MT activity in metal detoxification varies with different valence states of metal (Zeng et al., 2011; Yu et al., 2019) and various plant tissues such as roots or shoots (Yu et al., 2019). Numerous reports about the differential expression of plant MTs under metal stress are available, which functions in metal detoxification; for example, *Oryza sativa OsMT1b* and *OsMT2c* under Cr stress (Yu et al., 2019); *Oryza sativa OsMT1e* under Cd stress (Rono et al., 2021); *Physcomitrella patens PpMT2* under Cd and Cu stress (Liu Y. et al., 2020); and *Phytolacca americana PaMT3-1* under Cd stress (Zhi et al., 2020). On the other hand, in the case of ECM fungi, the differential expression of MTs: *LbMT1* and *LbMT2* in *Laccaria bicolor* under Cd and Cu stress, respectively (Reddy et al., 2014); *PaMT1* in *Pisolithus albus* under excess Cd and Cu stress (Reddy et al., 2016); HmMT3 in *Hebeloma mesophaeum* under Cd stress; and *SuiMT1* and *SuiMT2* in *Suillus indicus* under Cu stress (Shikha et al., 2019), leads to the detoxification of respective metal ions. The increased expression of symbiotic ECM MTs under metal stress causes the immobilization of metal ions in their extraradical mycelium and reduces the metal content in ECM roots compared to non-ECM roots (Reddy et al., 2016). Although not many studies are available regarding ECM effects on MT content of metal stressed host plants, past reports have determined the enhanced expression of host plant MTs upon their inoculation with arbuscular mycorrhizal (AM) fungi as compared to non-mycorrhizal plants under metal stress, ultimately leading to the host ameliorated metal tolerance (Cicatelli et al., 2010; Shabani and Sabzalian, 2016).

Glutathiones are well known to prevent oxidative stress and xenobiotics detoxification in cells (Sheehan et al., 2001). The upregulation of ECM fungal genes associated with glutathione biosynthesis under metal stress causes enhanced complexing of HM ions with glutathione and further sequestration of HM-glutathione complexes in their vacuoles. This process limits the HM transfer to their host plants, reducing HM toxicity (Khullar and Reddy, 2020). Similarly, the enhanced glutathione in ECM plants has been reported under metal stress compared to non-ECM plants, as shown in Table 1.

### Host Plant Tolerance Against Heavy Metal Stress-Induced Oxidative Burst

The excess concentration of plant nutrient Zn can lead to increased production of ROS, which, if it breaks its balance with ROS destruction, results in oxidative stress (Bartoli et al., 2013; Mohammadhasani et al., 2017). With evolution, plants acquire native defense mechanisms against oxidative stress, including antioxidant enzymes such as superoxide, catalase, glutathione peroxidase, ascorbate peroxidase, and guaiacol peroxidase (Wu et al., 2014). Plants under HM stress can increase their antioxidant activities as a defense mechanism, and mycorrhizal symbiosis further enhances this activity (Fernández-Fuego et al., 2017; Mohammadhasani et al., 2017). Catalase, peroxidase, ascorbate peroxidase, and superoxide dismutase are the antioxidants that play an essential role in alleviating the HM-induced oxidative stress in plants. The superoxide dismutase mediates the ROS species conversion to H_2_O_2_, whereas peroxidase and catalase mediate the H_2_O_2_ conversion to H_2_O (Li et al., 2015). The activities of these antioxidants in ECM fungi rise with increasing HM concentration upto certain levels, after which their activities decreases (Dachuan and Jinyu, 2021). The ECM symbiosis mediates the increase in antioxidant activities in their host plants against HM stress as shown by enhanced catalase, glutathione activity, and reduced malondialdehyde (MDA) content in metal stressed *Quercus acutissima* roots inoculated with *Suillus luteus* compared to non-ECM plants (Table 1; Sun et al., 2021). The lower MDA content corresponds to the stronger antioxidant activities of an organism. The reduction in MDA content of host *Pinus massoniana* under Al stress has also been reported upon their inoculation with *Suillus luteus* compared to non-ECM plants (Liu H. et al., 2020). It thus suggests the ECM-mediated improved antioxidant machinery of the host plant as one of the essential mechanisms for their enhanced HM tolerance.

### Alterations in the Nutrient Status of the Host Plants

The resource allocation between ECM fungal and plant partners strongly regulates the maintenance of long-term symbiotic association among both the partners. This long-term cooperation of nutrient transfer is maintained by transcriptional and translational regulations of transporter systems at regulatory checkpoints as shown in Figure 1 (Garcia et al., 2015). The presence of ECM symbiosis alters the transfer of nutrients from the soil to their host plants under HM stress (Table 2). The plant minerals such as Fe, Ca, N, P, and K content in roots and shoots get improved by ECM symbiosis in Pb-, Zn-, and Cd-contaminated soils (Hachani et al., 2020). The mycorrhiza-mediated N and P uptake in plants provides an ameliorated tolerance against oxidative stress (Begum et al., 2019). The P content is well correlated with the Cd concentration. The enhanced P content increases Cd accumulation in plants roots and significantly reduces Cd translocation upward in plants (Kong et al., 2020). At low Cd stress, the P content decreased in leaves and got doubled in the roots of ECM fungal plants. At high Cd stress, P content doubled in leaves and reduced in roots significantly compared with non-mycorrhizal plants under Cd stress, suggesting the vital role of ECM fungi in Cd retention of their host plants by regulating their P content (Sun et al., 2021). N and P enrichment can relieve Cd-induced oxidative stress in plants, possibly by increasing proline content (Kong et al., 2020). Proline acts as a potent non-enzymatic antioxidant and metal chelating agent (Sharma and Dietz, 2006).

**TABLE 2:** Alteration in the nutrition status of host plants by Ectomycorrhizal (ECM) symbiosis under metal stress.

ECM fungi	Host plant	Metal stress	Metal exposure period	Effects on ECM leaves	Effects on ECM roots	References
*Suillus luteus*	*Quercus acutissima*	Low Cd, 0.1 mg/kg	14 days	Ca, Mg, P, and K increased and Zn decreased	Ca, Mg, Zn and P increased; K decreased	Sun et al., 2021
*Suillus luteus*	*Quercus acutissima*	High Cd, 5 mg/kg	14 days	Ca, Mg, P, K, and Zn increased	K and P decreased	Sun et al., 2021
*Rhizopogon* sp.	*Pinus halepensis*	High Pb, Zn, and Cd stress	12 months	N, P, K, Ca, and Fe enhanced	N, P, K, Ca, and Fe Enhanced	Hachani et al., 2020
*Suillus luteus*	*Pinus sylvestris*	Zn, 0–1 mM	4 weeks	–	∙ Increase in Zn stress negatively relates to Fe content ∙ Low and high Zn stress enhances and reduced Ca accumulation, respectively	Zhang et al., 2021
*Pisolithus* sp., *Laccaria* sp., and *Cenococcum* sp.	*Pinus sylvestris*	Zn-, Cd-, and Pb-contaminated tailing pond soil	Pre-contaminated soil samples	Mg and Ca increased, whereas Fe content reduced	Pi, Mg, Ca, and Fe accumulation increased	Liu B. et al., 2020
*Pisolithus albus*	*Eucalyptus globulus* *Acacia spirorbis*	Ultramafic substrate	Pre-contaminated site	∙ N, P, K, and Ca increased ∙ Mg content decreased	-	Jourand et al., 2014

Further, the Na and P enrichment highly promotes Cd uptake and its sequestration with proline in plant roots which further decreases Cd transfer from sources to stem and helps to enhance the phytoextraction potential of the plant (Kong et al., 2020). On the other hand, the high external concentration of Zn in ECM plants negatively correlates with Fe accumulation in ECM roots. Ca uptake was enhanced under initial low external Zn and reduced under high Zn stress (Zhang et al., 2021). The antagonism between external Zn concentrations and Fe accumulation could be due to Fe displacement by Zn on ligand binding sites of metal transporters or siderophores (Suzuki et al., 2008; Hussein and Joo, 2019). Similarly, Langer et al. (2012) showed that the Fe uptake by ECM plants reduces under Zn stress compared to non-mycorrhizal plants, suggesting the competitive uptake among Fe, Zn, and Cd based on congruent ionic radii (Marschner, 1995). Among HM defense systems of ECM plants, the ion dilution effect is the mechanism under which the uptake and accumulation of nutrient minerals such as P and Mg got enhanced in plants to counteract the toxicity of harmful HMs to create HM dilution effects (Malcová et al., 2002; Canton et al., 2016). The increase in Ca and Mg content in ECM plants under HM stress suggests their role in improved plant biomass and further HM dilution effect in ECM plants as their tolerance mechanism (Sun et al., 2021).

On the other hand, *P. albus* symbiosis reduces Mg uptake in their host plants growing on ultramafic substrate rich in HMs to check excess Mg transfer. In contrast, host uptake for ultramafic soil deficient in N, P, K, and Ca increased, thus improving plant growth and creating a barrier for HMs present in excess (Jourand et al., 2014). The enhanced content of P, Mg, Ca, and Fe in *Pinus sylvestris* roots inoculated with different ECM fungal species under HM stress was reported compared to non-mycorrhizal plants. In shoots, reduced Fe content and increased Mg and Ca content were observed upon ECM symbiosis compared to non-mycorrhizal plants under metal stress (Liu B. et al., 2020). The varying uptake capacity of ECM plants for different nutrients could be due to the other kind of ECM fungi and their host plant species used and their tolerance potential for excess nutrients (Teotia et al., 2017).

At present, the restoration of abandoned mining lands is highly required to improve soil quality, microorganisms and plants growth for ecological rehabilitation (Wani et al., 2017). The use of traditional physical and chemical technologies for restoration programs can result in secondary pollution and high cost (Ayangbenro and Babalola, 2017).

The different species of ECM fungi affect host tolerance efficiency against metal stress differently (Sousa et al., 2012), thereby highlighting the need to optimize the best ECM fungal partner for the host before large-scale afforestation programs. In the reforestation of mine wastelands, the ECM infection rates in ECM fungal inoculated plant seedlings reduce after 6 months of their growth in mining lands (Chappelka et al., 1991; Hartley-Whitaker et al., 2000; Huang et al., 2012; Zong et al., 2015). The decline in ECM fungal colonization rate on land with high HM concentrations is associated with poor soil properties such as low organic matter, soil texture (Guo et al., 2007), macronutrients deficiency, and high processed residues content (Huang et al., 2012). The different species combination of ECM fungi and host plants gives different mycorrhization rates (Zong et al., 2015). On Pb-, Zn-, and Cd-polluted land, ECM fungal community richness and diversity have been correlated with soil N content but not with Pb, Zn, and Cd concentrations. For example, the dominant ECM fungal species obtained on N deficient tailing are mostly found on N-poor soils (Huang et al., 2012). By contrast, the diversity of ECM fungal community associated with white oak and Mason pine on Mn mine site correlated with less toxic Mn concentration (Huang et al., 2014). The Cd hyperaccumulator ecotypes of *Sedum alfredii* have more Cd accumulation in roots than non-hyperaccumulators (Sun et al., 2013). Among the different strains of the same ECM fungal species, the HM tolerant ecotypes of species are reported to be more potent for enhancing metal tolerance and growth in their host plants. Hence, this depicts ECM fungal selection’s importance for the phytoremediation programs of HM contaminated lands (Szuba et al., 2017). It is necessary to focus future studies on determining the ECM-mediated antagonist or synergistic effects of external HM stress with the dynamics of different nutrients in host plants, which may participate in plant response to HM stress. As different species of ECM fungi affect plant HM defense systems differently, further studies are required to explore the factors playing a role behind this. Furthermore, plant phytohormones such as auxin, ethylene, and abscisic acid are widely reported for their important roles in plant defense response against HM stress conditions (Emamverdian et al., 2021). Inoculating AM fungi to *Robinia pseudoacacia* seedlings under As stress enhanced the Indole-3-acetic acid and abscisic acid content, decreasing the zeatin riboside gibberellic acid concentrations and altering the ratios of various phytohormones in the host plant. These results suggest that the mycorrhiza-mediated phytohormones are essential factors behind host-alleviated metal toxicity (Zhang et al., 2020). Although, exogenously applying phytohormones and manipulating the plant’s endogenous level of phytohormones are reported as promising ways to enhance plants tolerance against metal stress (Saini et al., 2021). Several past studies reported that the ECM symbiosis significantly alters the hormonal status of their host plants (Felten et al., 2009; Basso et al., 2020). It would be interesting to explore the impact of ECM inoculation on hormones derived metal tolerance in host plants, which is not yet extensively reported. Besides the few lighter elements possessing naturally occurring radioisotopes, all elements with an atomic number more than 83 are considered radioactive (Thompson et al., 1949). To these natural radionuclides, several anthropogenic activities, such as nuclear industries, mining, and nuclear weapon trials, further add up radionuclides concentration in environment (Hain et al., 2020). The bioavailability and mobility of radionuclides significantly influence their deleterious impacts on environment and human health (Lopez-Fernandez et al., 2021). The AM symbiosis significantly affects the transfer of soil ribonuclides to their host plants. When inoculated with the same host plant, further ribonuclease retention in roots or shoot transfer varies depending upon different AM fungal species. For bioremediation purpose, the utilization of mycorrhizal symbiosis in enhancing phytoaccumulation of ribonuclides requires further studies (Rosas-Moreno et al., 2021). Similarly, the impact of ECM symbiosis over radionuclides accumulation in their host plants is reported in a few studies but remains unclear and needs focus studies (Ogo et al., 2018).

## Conclusion

The ECM symbiosis improves the HM tolerance and growth of their metal stressed host plants through several mechanisms, which thus help in regenerating the metal-contaminated lands with plants. First, the ECM fungi can either act as a physical barrier between soil HM and plant roots or enhance HM accumulation in host plants depending on several factors such as HM type, external concentrations, and plant and fungal species. ECM fungi can also prevent HM transfer to plant roots by sequestering them on fungal cell walls or intracellularly with MTs and glutathione. ECM symbiosis ameliorates plant growth and their tolerance to oxidative stress under HM stress. The ECM fungi can change the nutrition dynamic of plants with soil in such a way to create HM dilution effects and to prevent HM transfer from roots to shoots. The role of ECM promoted or inhibited release plant root exudates in HM stress tolerance needs more studies for clarification. On the basis of the collected evidences, the ECM symbiosis proves to be beneficial for promoting plant growth on metal-contaminated lands and enhancing soil HM phytoextraction in host plants to reduce soil HM content. This field requires more extensive studies to understand the nutrient dynamics of ECM plants under metal stress and how it affects their tolerance.

## Author Contributions

EC conducted the literature review and designed and wrote the manuscript. MR supervised, corrected, improved, and accepted the final version of the manuscript. Both authors contributed to the article and approved the submitted version.

## Conflict of Interest

The authors declare that the research was conducted in the absence of any commercial or financial relationships that could be construed as a potential conflict of interest.

## Publisher’s Note

All claims expressed in this article are solely those of the authors and do not necessarily represent those of their affiliated organizations, or those of the publisher, the editors and the reviewers. Any product that may be evaluated in this article, or claim that may be made by its manufacturer, is not guaranteed or endorsed by the publisher.

## Abbreviations

Al: Aluminium
Fe: Iron
Ni: Nickel
Pb: Lead
Zn: Zinc
Cd: Cadmium
H: Hydrogen
C: Carbon
Cu: Copper
Cr: Chromium
Ca: Calcium
K: Potassium
P: Phosphorus
N: Nitrogen
Na: Sodium
Mg: Magnesium
Mn: Manganese.
